# Mendelian Randomization Analysis Identifies Blood Tyrosine Levels as a Biomarker of Non-Alcoholic Fatty Liver Disease

**DOI:** 10.3390/metabo12050440

**Published:** 2022-05-13

**Authors:** Émilie Gobeil, Ina Maltais-Payette, Nele Taba, Francis Brière, Nooshin Ghodsian, Erik Abner, Jérôme Bourgault, Eloi Gagnon, Hasanga D. Manikpurage, Christian Couture, Patricia L. Mitchell, Patrick Mathieu, François Julien, Jacques Corbeil, Marie-Claude Vohl, Sébastien Thériault, Tõnu Esko, André Tchernof, Benoit J. Arsenault

**Affiliations:** 1 Centre de Recherche de l’Institut Universitaire de Cardiologie et de Pneumologie de Québec, Québec, QC G1V 4G5, Canada; emilie.gobeil.2@ulaval.ca (É.G.); ina.maltais-payette.1@ulaval.ca (I.M.-P.); maria.ghodsian@gmail.com (N.G.); jerome.bourgault@criucpq.ulaval.ca (J.B.); eloi.gagnon.1@ulaval.ca (E.G.); hasanga.manik-purage.1@ulaval.ca (H.D.M.); christian.couture@kin.ulaval.ca (C.C.); patricia.mitchell@criucpq.ulaval.ca (P.L.M.); patrick.mathieu@fmed.ulaval.ca (P.M.); francjulien@me.com (F.J.); sebastien.theriault@criucpq.ulaval.ca (S.T.); andre.tchernof@criucpq.ulaval.ca (A.T.); 2 Estonian Genome Center, Institute of Genomics, University of Tartu, Riia 23b, 51010 Tartu, Estonia; nele.taba@ut.ee (N.T.); erik.abner@ut.ee (E.A.); tonu.esko@ut.ee (T.E.); 3 Institute of Molecular and Cell Biology, University of Tartu, Riia 23, 51010 Tartu, Estonia; 4 Centre de Recherche du CHU de Québec, Québec, QC G1V 4G2, Canada; francis.briere@crchudequebec.ulaval.ca (F.B.); jacques.corbeil@crchudequebec.ulaval.ca (J.C.); 5 Department of Surgery, Faculty of Medicine, Université Laval, Québec, QC G1V 0A6, Canada; 6 Department of Molecular Medicine, Faculty of Medicine, Université Laval, Québec, QC G1V 0A6, Canada; 7 Centre NUTRISS, Institut sur la Nutrition et les Aliments Fonctionnels, Université Laval, Québec, QC G1V 0A6, Canada; marie-claude.vohl@fsaa.ulaval.ca; 8 School of Nutrition, Université Laval, Québec, QC G1V 0A6, Canada; 9 Department of Molecular Biology, Medical Biochemistry and Pathology, Faculty of Medicine, Université Laval, Québec, QC G1V 0A6, Canada; 10 Department of Medicine, Faculty of Medicine, Université Laval, Québec, QC G1V 0A6, Canada

**Keywords:** non-alcoholic fatty liver disease, tyrosine, biomarker, metabolites, obesity, Mendelian randomization

## Abstract

Non-alcoholic fatty liver disease (NAFLD) is a complex disease associated with premature mortality. Its diagnosis is challenging, and the identification of biomarkers causally influenced by NAFLD may be clinically useful. We aimed at identifying blood metabolites causally impacted by NAFLD using two-sample Mendelian randomization (MR) with validation in a population-based biobank. Our instrument for genetically predicted NAFLD included all independent genetic variants from a recent genome-wide association study. The outcomes included 123 blood metabolites from 24,925 individuals. After correction for multiple testing, a positive effect of NAFLD on plasma tyrosine levels but not on other metabolites was identified. This association was consistent across MR methods and was robust to outliers and pleiotropy. In observational analyses performed in the Estonian Biobank (10,809 individuals including 359 patients with NAFLD), after multivariable adjustment, tyrosine levels were positively associated with the presence of NAFLD (odds ratio per 1 SD increment = 1.23 [95% confidence interval = 1.12–1.36], *p* = 2.19 × 10^−5^). In a small proof-of-concept study on bariatric surgery patients, blood tyrosine levels were higher in patients with NAFLD than without. This study revealed a potentially causal effect of NAFLD on blood tyrosine levels, suggesting it may represent a new biomarker of NAFLD.

## 1. Introduction

Non-alcoholic fatty liver disease (NAFLD) is the most common liver disease, with an estimated prevalence of one in four adults in most Western countries [1]. NAFLD is a progressive disease initiated by the accumulation of lipid droplets within hepatocytes which can lead to inflammation, cell death and to more advanced stages such as non-alcoholic steatohepatitis (NASH) (with or without fibrosis), cirrhosis and liver cancer. Cardiovascular diseases are the leading cause of death in patients with NAFLD [2]. This condition is also associated with other comorbidities such as type 2 diabetes, chronic kidney disease and gastrointestinal neoplasms [3,4,5]. There is currently no pharmacological treatment available specifically for the treatment of NAFLD.

According to the National Institutes of Health U.S. National Library of Medicine, there are currently more than 300 ongoing randomized clinical trials (RCTs) enrolling patients with NAFLD. Such RCTs are challenging because NAFLD “diagnosis” often requires invasive methods and/or imaging approaches, which are clinically burdensome and cost-prohibitive, especially since NAFLD has reached epidemic proportions in developing countries that may not have the clinical, financial, and infrastructural resources to identify and adequately treat patients with NAFLD. For example, liver biopsy is not only invasive and expensive but is also prone to sampling error [6]. Affordable and easily obtainable tests are required to identify NAFLD patients who may benefit from therapies under investigation. Blood biomarkers of NAFLD that are not modulated by secondary non-causal pathways may be promising candidates for the identification of at-risk individuals and for the development of tailored therapy for NAFLD.

Mendelian randomization (MR) is a modern epidemiology investigation technique that is increasingly used to explore whether risk factors associated with disease traits reflect true causal associations or not [7]. Akin to an RCT, MR takes advantage of the random allocation of genetic variation at conception to explore whether human traits that are at least in part under genetic control are associated with diseases. MR has also been used to determine whether a genetic susceptibility to certain diseases influences other biological traits such as the blood metabolome, thereby identifying early biomarkers of disease-related traits [8,9].

High-quality MR studies rely on the availability of standardized effect sizes of the major genetic variants associated with a trait of interest when that trait is used as an exposure, and on the availability of genome-wide association study (GWAS) summary statistics when that trait is used as an outcome. In a recent study [10], we performed a GWAS meta-analysis of NAFLD in four cohorts totaling 8434 NAFLD cases and 770,180 controls. This analysis identified genetic variants at or near the *GCKR, LPL, TRIB1*, *FTO, MAU2/TM6SF2, APOE* and *PNPLA3* as NAFLD-susceptibility loci. In this study, we used a combination of observational and two-sample MR study designs to identify blood metabolites that may be causally influenced by the presence of NAFLD.

## 2. Results

### 2.1. Mendelian Randomization Analysis on the Impact of Non-Alcoholic Fatty Liver Disease on the Blood Metabolome

**Figure 1:**
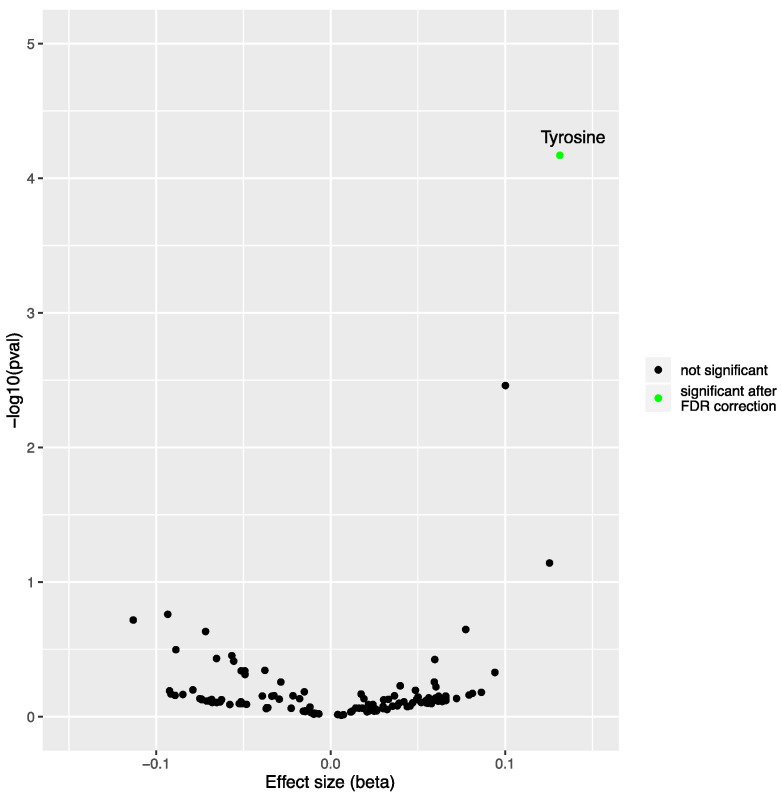
Causal impact of genetically predicted non-alcoholic fatty liver disease (NAFLD) using seven genome-wide significant SNPs on the blood metabolome. Volcano plot depicting the effect of genetically predicted NAFLD on blood metabolites (*n* = 123) using inverse-variance weighted Mendelian randomization. Each dot represents a different metabolite, and the green dot represents the metabolite (here, tyrosine) significantly influenced by the presence of NAFLD following correction for false-discovery rate (pFDR < 0.05/123 metabolites).

**Figure 2:**
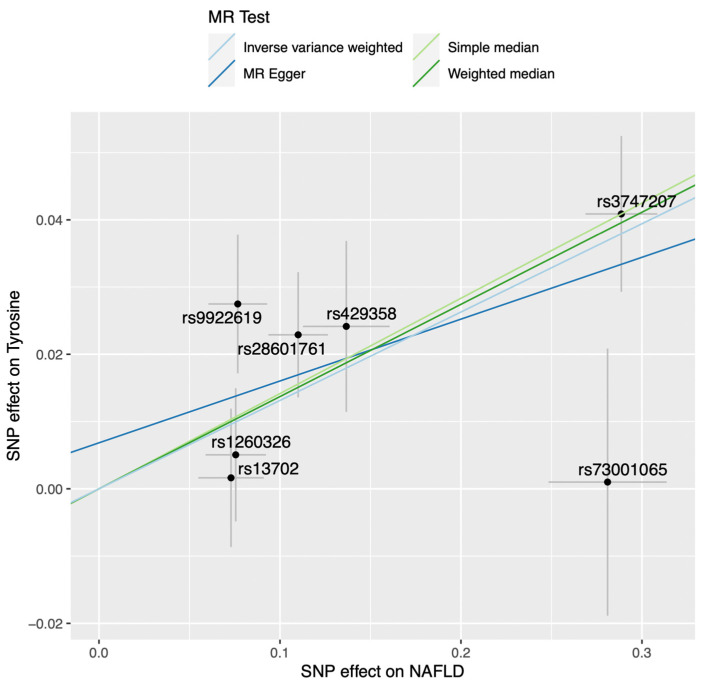
A Mendelian randomization study of genetically predicted non-alcoholic fatty liver disease and plasma levels of tyrosine. Scatter plot showing the estimated effect sizes of each of the seven genetic loci associated with NAFLD on NAFLD and blood tyrosine levels and the regression slopes of four MR methods (inverse-variance weighted, simple median, weighted median, and MR-Egger).

First, we explored the potentially causal effect of genetically predicted NAFLD on blood metabolites, using genetic instruments in instrumentally variable analysis implemented via two-sample MR. To perform two-sample MR, we used the summary statistics of two GWAS: NAFLD (exposure) and 123 metabolites (outcome). Our genetic instrument included lead variants at each of seven NAFLD-susceptibility loci (F-statistic = 61). Using inverse-variance weighted (IVW) MR, we found that genetically predicted NAFLD was robustly associated with higher levels of tyrosine (*p* = 6.75 × 10^−5^) after correction for false-discovery rate with the Benjamini–Hochberg method (pFDR < 4.06 × 10^−4^ [0.05/123 metabolites]) (Figure 1). We also found an association between NAFLD and the tyrosine precursor phenylalanine (*p* = 0.0035, Supplementary Table S2), although this association did not pass the FDR-corrected statistical significance threshold. The association between NAFLD and tyrosine levels was consistent across MR methods and robust to outliers and pleiotropy (Table 1 and Figure 2). Because there was sample overlap for 3287 individuals between the exposure (genetically predicted NAFLD) and outcomes (blood metabolites), with the Estonian Biobank contributing to both datasets, we performed another NAFLD GWAS meta-analysis, which excluded the Estonian Biobank participants (4119 NAFLD cases and 190,129 controls). Genetically predicted NAFLD was still associated with tyrosine (beta [SE] = 0.085 [0.026], *p* = 9.37 × 10^−4^) using IVW-MR (data not shown). One of the key assumptions of MR is that genetic variants used as a proxy of the exposure influence the outcome via their effect on the exposure and not via other related traits (horizontal pleiotropy) [7,11]. Our original NAFLD GWAS identified seven NAFLD-susceptibility loci [10]. Some of these loci were identified after leveraging prior effect sizes of NAFLD-related traits such as body mass index and triglycerides, which might increase the chance of finding associations that may be influenced by NAFLD-related traits and not by NAFLD per se. We therefore investigated the impact of genetically predicted NAFLD on blood levels of tyrosine using independent NAFLD SNPs from our original GWAS. For that purpose, we used 12 NAFLD-associated SNPs with *p*-value for association < 5 × 10^−6^ and a r2 < 0.001. Multiple MR methods were used to investigate the association of genetically predicted NAFLD using the 12-SNP instruments (F-statistic = 47) with tyrosine levels. The independence of genetic instruments was ensured by obtaining the LD matrix using the European 1000-genome LD reference panel (Supplementary Table S4). Leave-one-out analysis confirmed that the results were robust to the presence of outliers (Supplementary Table S3). Results presented in Table 1 suggest that genetically predicted NAFLD was strongly associated with tyrosine levels using this other genetic instrument that might be less susceptible to horizontal pleiotropy.

### 2.2. Observational Analysis of the Impact of Non-Alcoholic Fatty Liver Disease on Blood Tyrosine Levels in the Estonian Biobank

We next investigated whether the presence of NAFLD was associated with higher plasma levels of tyrosine in the Estonian Biobank. Tyrosine levels were measured in 10,809 individuals including 359 patients with NAFLD (obtained from electronic health records). Table 2 presents the association between tyrosine levels per one standard-deviation increment and NAFLD before and after multivariable adjustment. After adjusting for age, sex, smoking, education, and BMI, tyrosine levels were positively associated with the presence of NAFLD (odds ratio per 1 SD increment = 1.23 [95% confidence interval = 1.12–1.36, *p* = 2.19 × 10^−5^]). Altogether, these results provide validation from an observational study of the association of NAFLD with plasma tyrosine levels.

### 2.3. Impact of Non-Alcoholic Steatohepatitis on Tyrosine Levels in Patients Undergoing Bariatric Surgery

**Figure 3:**
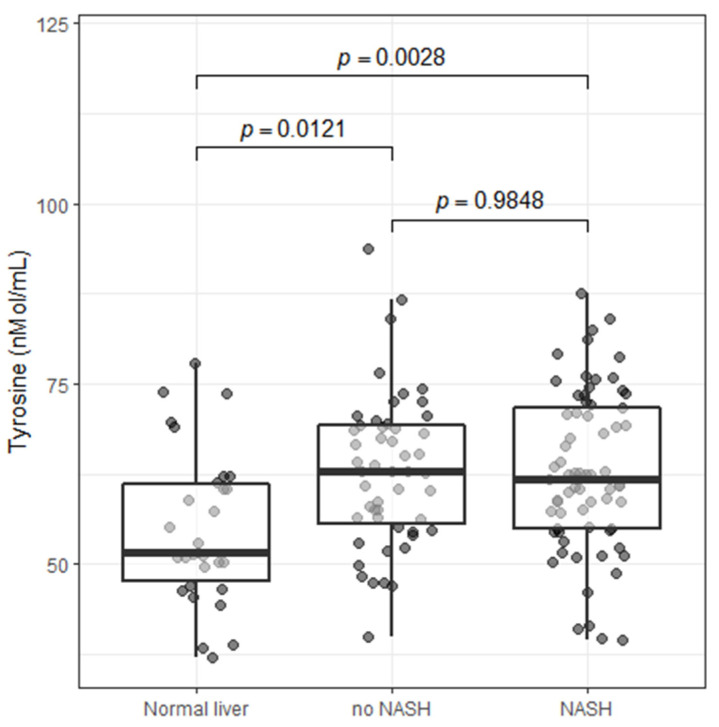
Observational analysis of the impact of NAFLD and NASH on tyrosine levels in the IUCPQ Obesity Biobank. Box plot representing the dispersion and the median values of tyrosine levels between the three groups (without NAFLD = 54.9 ± 10.7 nmol/mL; with NAFLD–without NASH = 62.5 ± 10.9 nmol/mL; with NAFLD and NASH = 62.9 ± 11.0 nmol/mL). *p*-values are from Tukey HSD test.

Although elevated body weight is an important risk factor for NAFLD, the presence and severity of NAFLD is very heterogeneous among patients with obesity. Whether blood-based biomarkers of NAFLD such as tyrosine levels could help identify and stratify patients with NAFLD/NASH is unknown. We therefore investigated in a small proof-of-concept study whether tyrosine levels were associated with the presence of NAFLD with or without histologically confirmed NASH among 138 participants of the IUCPQ Obesity Biobank. Supplementary Table S6 presents the clinical information at the time of surgery for patients of each group (healthy liver, NAFLD without or with NASH). Compared to patients without NAFLD (*n* = 30), blood tyrosine levels were higher in those with NAFLD (without NASH (*n* = 39) or with NASH (*n* = 69)). However, among patients with NAFLD, tyrosine levels were comparable among patients with or without NASH (Figure 3). These results suggest that plasma tyrosine levels could be useful to identify patients with NAFLD among patients with obesity, but it may not identify patients with a more advanced stage of NAFLD such as NASH.

## 3. Discussion

We established an MR framework aimed at identifying novel biomarkers of NAFLD. Results of this analysis suggest that genetically predicted NAFLD may not be causally linked with metabolites associated with triglyceride-rich lipoprotein metabolism, glucose-insulin homeostasis, or branched-chain amino acid levels. However, this MR analysis revealed an effect of NAFLD on blood tyrosine levels, which may represent a new clinical biomarker of NAFLD. We also reported that patients with higher blood levels of tyrosine had a higher prevalence of NAFLD in the Estonian Biobank, and that among patients with obesity, blood tyrosine levels were higher in patients with NAFLD. Results from the two observational studies with different clinical settings (i.e., a population-based biobank and a bariatric surgery cohort), provide validation to our initial findings obtained with MR.

Several observational studies have suggested that liver fat accumulation or NAFLD negatively impacts triglyceride-rich lipoprotein metabolism, glucose–insulin homeostasis as well as branched-chain amino acid levels [12,13,14,15,16]. We investigated whether the presence of NAFLD impacted lipoprotein levels and metabolites of these pathways to identify early biomarkers of NAFLD using MR. This analysis did not find evidence of a causal association of NAFLD with triglyceride-rich lipoprotein metabolism, which is expected since some variants were associated with higher lipid levels while other variants were associated with lower lipid levels. NAFLD was not however associated with glucose–insulin homeostasis markers or branched-chain amino acids. We did however find a significant impact of NAFLD on tyrosine and, to a lesser extent, its metabolic precursor phenylalanine. Although the impact of NAFLD on tyrosine metabolism was reported decades ago [17], our analysis adds to this body of evidence by suggesting that the impact of NAFLD on tyrosine metabolism might be a direct consequence of NAFLD, and that this association might not be driven by secondary causes of NAFLD.

Previous studies have investigated the link between excess adiposity and its metabolic consequences, such as NAFLD and levels of amino acids such as tyrosine. A study by Kimberley et al. provided evidence that tyrosine was positively related to waist circumference (WC) and body mass index (BMI) in 997 participants of the Framingham cohort [18]. Another study performed in a cohort of 38 participants in an outpatient clinic (separated into four groups: 10 patients with normal glucose tolerance and NAFLD, 10 patients with T2D and NAFLD, eight patients with T2D and no liver disease and a group of 10 controls) identified several non-branched-chain amino acids, including tyrosine, that may be increased in patients with NAFLD without T2D [19]. In two other metabolomic studies, Boulet et al. and Brennan et al. showed that tyrosine was positively related to many adiposity indices including abdominal fat cell size and adipose tissue depots [20,21]. Boulet et al. tested the association between levels of 138 metabolites detectable in plasma and adiposity measurements in 59 healthy women. Concentrations of tyrosine were positively associated with visceral adipose tissue area and subcutaneous adipose tissue area and were significantly associated with the mean adipocyte diameter in both fat compartments. In a study of 103 middle-aged patients with abdominal obesity, Brennan et al. reported significant associations between tyrosine levels and abdominal adipose tissue and between visceral adipose tissue accumulation and phenylalanine, the precursor of tyrosine. Whether these associations could be explained by the fact that NAFLD causes elevations in tyrosine levels, which in turn influence the fate of adipocytes, needs to be further explored. Furthermore, these studies suggest a disruption of the hepatic amino acid metabolism in the setting of NAFLD, but the mechanisms underlying the relationship between amino acids’ imbalances is poorly understood. Winther-Sørensen et al. recently reported that individuals with hepatic steatosis had impaired clearance of amino acids [22]. However, tyrosine was not considered in this investigation. In another study, individuals with NAFLD were also characterized by higher gene expression of metabolic enzymes that may influence amino acid release into circulation [23].

Our study has limitations. For instance, although we used the largest NAFLD dataset available to date and have excluded secondary causes of NAFLD whenever possible, an electronic health records-based diagnosis of complex diseases such as NAFLD might be prone to misclassification of cases and controls. The prevalence of NAFLD was also not available in some of the cohorts used to document the impact of NAFLD on the blood metabolome (24,925 individuals from 10 European cohorts). MR yields precise causal estimates under three core assumptions. The values of the F-statistic indicated sufficient strength for the seven SNPs instruments (F-statistic = 61) and for the 12 SNPs instruments (F-statistic = 47). The exchangeability assumption, knowns as the second assumption, states that the instruments should have no impact on confounders (i.e., exert no horizontal pleiotropic effect). Our results are likely robust to the presence of pleiotropy, since robust MR analyses returned consistent causal estimates. Additionally, different techniques were used for the quantification of tyrosine levels, but this does not affect the interpretation of the results. Results from the IUCPQ Obesity Biobank also need to be interpreted with caution given the small sample size. Residual confounding cannot be excluded since participants were not carefully matched, and a selection bias might stem from the fact that the study sample was not randomly selected. The complex biology of NAFLD makes it difficult to understand the underlying molecular biology or exact pathways involved, and this can introduce bias in interpretation of the results. Studies documenting the impact of NAFLD resolution on tyrosine levels could also consolidate the causal effect of NAFLD on the blood metabolome.

In conclusion, our study identified a blood metabolite, the amino acid tyrosine, that may be causally influenced by the presence of NAFLD. These findings shed light on the metabolic consequences of NAFLD but also identify a potential early biomarker of NAFLD that could be used to identify patients who may benefit from therapies targeting NAFLD and/or for risk stratification in this population. Additional studies will be required to determine whether our findings could be helpful to optimizing NAFLD risk prediction as well as patient recruitment for trials aiming at preventing and/or treating NAFLD.

## 4. Materials and Methods

### 4.1. Mendelian Randomization Analyses on the Impact of NAFLD on the Blood Metabolome

To perform the MR analysis, we combined information of two publicly available GWAS summary statistics in a two-sample MR setting. Genetic association estimates for NAFLD (exposure) were obtained from our recently published GWAS [10] (8434 cases and 770,180 controls) of European ancestry from four cohorts. Briefly, we performed a fixed-effect GWAS meta-analysis of the Estonian Biobank, the UK Biobank, The Electronic Medical Records and Genomics (eMERGE) [24] network, and FinnGen with the *METAL* package [25]. NAFLD cases were obtained by electronic health record codes or hospital records. For the logistic regression analysis, we performed an adjustment for age, sex, genotyping site and the first three ancestries based on the principal components. For MR analysis, we selected the seven lead SNPs, associated with NAFLD through risk-factor-informed GWAS, for each risk locus (at or near the *GCKR*, *LPL*, *TRIB1*, *FTO*, *MAU2/TM6SF2*, *APOE* and *PNPLA3* loci) (Supplementary Table S1). Additional information about the selected variants from the GWAS for NAFLD and how they were assigned to genes are available in the original article [10]. For the selection of the 12 SNPs instrument of genetically predicted NAFLD (Results), SNPs with a *p* ≤ 5 × 10^−6^ were kept. We ensured the independence of genetic instruments by clumping all neighbouring SNPs into a 10 Mb window with a linkage disequilibrium r2 < 0.001 using the European 1000-genome LD reference panel (Supplementary Table S4). The strength of the instruments for NAFLD was evaluated with the Cragg–Donald F-statistic [26] for an effective sample size of 16,685 participants [27]. We used GWAS summary statistics from the study of Kettunen et al. [28] to define our study outcomes. In this study, 123 blood lipids and metabolites were measured in 24,925 individuals from 10 European cohorts using high-throughput nuclear magnetic resonance spectroscopy. Metabolites measured using this platform represent a broad molecular signature of systemic metabolism and include metabolites from multiple metabolic pathways (mostly lipoprotein lipids and subclasses, fatty acids and amino acids, glycolysis precursors). The estimates of the metabolites were normalized and reported on a standard deviation scale. The association between genetically determined NAFLD (first with seven SNPs and then with 12 SNPs) and the blood metabolome was assessed using the IVW-MR with the *mr* function from the *TwoSampleMR* package in R [7]. The IVW-MR is comparable to performing a meta-analysis of each Wald ratio (the effect of the genetic instrument on the outcome divided by its effect on the exposure). Additional MR analysis were performed to evaluate horizontal pleiotropy (intercept *p*-value from MR Egger [29]) and the presence of outliers. We used MR-PRESSO [30], an outlier-robust method, to detect the presence of outliers (variants potentially causing pleiotropy and influencing causal estimates) and causal estimates were obtained before and after excluding outliers. We also used, as sensitivity analyses, the simple median and weighted median consensus methods, which give unbiased causal inference if most genetic instruments are valid. Altogether, consistent results across different robust MR methods and significant *p*-values after correction for multiple testing give support to the robustness and provide further confirmation of the nature of the causal finding.

### 4.2. Impact of NAFLD on Tyrosine Levels in the Estonian Biobank

Blood plasma levels of tyrosine were measured using nuclear magnetic resonance spectroscopy on 10,809 participants of the Estonian Biobank. The Estionan Biobank is a population-based biobank that is longitudinal with periodic updates from the e-Health databases (EMR), including ICD-10 codes, from National Health Insurance Fund, prescription data, laboratory data, infraction registry, cancer registry data, causes of death registry, regional hospital databases, research projects, national registries and databases for enrichment of phenotype data in the Estonian Biobank. All participants provided written informed consent. Metabolomic nuclear magnetic resonance (NMR) is available for 120 molecules, including tyrosine, and was measured among 11,000 individuals. These participants were also included in the GWAS meta-analysis of NAFLD that was used to generate the study exposure in the MR analysis. Two groups were formed, participants without NAFLD (*n* = 10,450) and for those with NAFLD (*n* = 359), based on their electronic health records. Clinical characteristics of participants are presented in Supplementary Table S5. Odds ratios and corresponding *p*-values were estimated using a logistic regression model implemented in R version 4.0.4 [31]. Metabolite values were scaled and centered prior to analysis. Two models were run: a raw model with adjustment for age and sex, and an adjusted model, which was additionally adjusted for smoking status, education level and body-mass index.

### 4.3. Impact of NAFLD and NASH on Tyrosine Levels in Québec Bariatric Surgery Cohort

In a proof-of-concept study, plasma tyrosine levels were measured in sample of 138 participants from the IUCPQ Obesity Biobank according to institutionally approved management modalities. The IUCPQ Obesity Biobank includes liver biopsies of patients who have undergone a bariatric surgery at the Institut universitaire de cardiologie et de pneumologie de Québec and who agreed to contribute to the biobank. All participants provided written, informed consent. Histological lesions from liver biopsy were graded and staged using the criteria of Brunt [32], by pathologists who were blinded to the study objectives. Since few patients from this biobank have no liver steatosis, we identified patients without liver steatosis (*n* = 30) and patients with various severities of NAFLD that have similar characteristics regarding age, sex, ethnicity, BMI, etc., and based on the following three categories: (1) absence of hepatic steatosis (*n* = 30), (2) hepatic steatosis without NASH (*n* = 39) and (3) hepatic steatosis with NASH (*n* = 69). Supplementary Table S6 presents the clinical characteristics at the time of surgery (sex, age, anthropometry, medication use, and glycaemic, lipoprotein and liver enzyme profiles) for patients of each group (without NAFLD, NAFLD without NASH and NAFLD with NASH). Plasma levels of tyrosine were quantified using a Water Acquity UPLC system coupled to a Synapt G2-Si mass spectrometer (Waters, Milford, MA, USA) in tandem mode (LC-MS/MS) using the EZ:faast amino acid sample testing kit (Phenomenex, 2003, Torrance, CA, USA). With this kit, plasma samples are mixed with an internal standard solution and amino acids are extract by a solid-phase support. Once extracted, amino acids are derivatized to increase their stability during analysis and purified by a two-phase liquid–liquid extraction. The samples were analyzed with an HPLC column, method gradient, and multiple monitoring (MRM), which were provided by the kit. For the last step of the quantification, sample-to-internal-standard ratios and a five-point calibration curve ranging from 20 nmol/mL to 200 nmol/mL were used. An analysis of variance (ANOVA) followed by Tukey HSD test were performed to compare mean tyrosine levels between the three groups.

### 4.4. URLs

- GWAS summary statistics for NAFLD (study accession: GCST90091033) are available at: https://www.ebi.ac.uk/gwas/studies/GCST90091033 (accessed on 30 August 2021).
- GWAS summary statistics for the 123 metabolites are available on MR-Base GWAS Catalog (GWAS id: “met-c-838” to “met-c-960”): https://gwas.mrcieu.ac.uk/datasets/?gwas_id__icontains=met-c (accessed on 30 August 2021). Additional information about the metabolites can be accessed in the article from Kettunen et al., 2016 (Supplementary Table S4): https://www.ncbi.nlm.nih.gov/pmc/articles/PMC4814583/ (accessed on 30 August 2021).
- The TwoSampleMR package is available at: https://github.com/MRCIEU/TwoSampleMR (accessed on 30 August 2021).
- The MendelianRandomization package is available at: https://github.com/cran/MendelianRandomization (accessed on 30 August 2021).
- The data.table package is available at https://github.com/Rdatatable/data.table (accessed on 30 August 2021).
- The tidyverse package collection is available at: https://github.com/tidyverse/tidyverse (accessed on 30 August 2021).
- The LDlinkR package is available at: https://github.com/CBIIT/LDlinkR (accessed on 30 August 2021).

## Figures and Tables

**Table 1:** Association of genetically predicted non-alcoholic fatty liver disease with blood tyrosine levels across multiple Mendelian randomization methods.

N SNPs	Inverse-Variance Weighted			Simple Median			Weighted Median			MR-Egger		MR PRESSO
	Beta	SE	*p*-Value	Beta	SE	*p*-Value	Beta	SE	*p*-Value	Intercept	*p*-Value Intercept	Outlier Test *p*-Value
7	0.131	0.033	6.75 × 10^−5^	0.142	0.045	0.002	0.137	0.038	2.82 × 10^−4^	0.010	0.515	0.334
12	0.104	0.027	1.47 × 10^−4^	0.042	0.040	0.297	0.103	0.036	3.88 × 10^−3^	−0.002	0.759	0.293

The effect of genetically predicted non-alcoholic fatty liver disease on tyrosine levels using two genetic instruments (one with seven genome-wide significant variants and one with the 12 independent NAFLD variants with *p*-value for NAFLD associations < 5 × 10^−6^ are presented.

**Table 2:** Association of plasma tyrosine levels (SD) with the presence of non-alcoholic fatty liver disease in the Estonian Biobank.

Tyrosine	Odds Ratio (95% CI) for NAFLD Presence in the Estonian Biobank *	*p*-Value
Model 1	1.29 (1.18–1.42)	2.09 × 10^−8^
Model 2	1.23 (1.12–1.36)	2.19 × 10^−5^

Model 1 is adjusted for age and sex. Model 2 is adjusted for age, sex, smoking, education and body-mass index. * OR per 1 SD increase in tyrosine levels.

## Data Availability

All genome-wide association study summary statistics used in this study are publicly available. All data needed to evaluate this work are present in the paper and/or the Supplementary Materials. Accession number and URLs are provided in the methods section. Additional data related to the current study are not publicly available but are available from the corresponding author upon reasonable request.

## Supplementary Materials

The following supporting information can be downloaded at: www.mdpi.com/article/10.3390/metabo12050440/s1, Table S1: Instruments for genetic-predicted NAFLD (7 and 12 SNPs); Table S2: Association of non-alcoholic fatty liver (predicted by a genetic instrument that includes all independent SNP associated with NAFLD at the *p* < 5 × 10^−8^ threshold) with blood metabolites across IVW Mendelian randomization method; Table S3: Leave-one-out MR analysis results; Table S4: LD matrix for the 7 variants of NAFLD; Table S5: Clinical characteristics of participants of the Estonian Biobank cohort; Table S6: Clinical characteristics of participants of the IUCPQ Obesity Biobank cohort.

## References

[1] Younossi Z.M., Koenig A.B., Abdelatif D., Fazel Y., Henry L., Wymer M. (2016). Global epidemiology of nonalcoholic fatty liver disease—Meta-analytic assessment of prevalence, incidence, and outcomes. Hepatology.

[2] Targher G., Byrne C.D., Tilg H. (2020). NAFLD and increased risk of cardiovascular disease: Clinical associations, pathophysiological mechanisms and pharmacological implications. Gut.

[3] Byrne C.D., Targher G. (2015). NAFLD: A multisystem disease. J. Hepatol..

[4] Armstrong M.J., Adams L.A., Canbay A., Syn W.-K. (2014). Extrahepatic complications of nonalcoholic fatty liver disease. Hepatology.

[5] Masoodi M., Gastaldelli A., Hyötyläinen T., Arretxe E., Alonso C., Gaggini M., Brosnan J., Anstee Q.M., Millet O., Ortiz P. (2021). Metabolomics and lipidomics in NAFLD: Biomarkers and non-invasive diagnostic tests. Nat. Rev. Gastroenterol. Hepatol..

[6] Estep J., Birerdinc A., Younossi Z. (2010). Non-invasive diagnostic tests for non-alcoholic fatty liver disease. Curr. Mol. Med..

[7] Hemani G., Zheng J., Elsworth B., Wade K.H., Haberland V., Baird D., Laurin C., Burgess S., Bowden J., Langdon R. (2018). The MR-Base platform supports systematic causal inference across the human phenome. (Clinical report). eLife.

[8] Mohammadi-Shemirani P., Sjaarda J., Gerstein H.C., Treleaven D.J., Walsh M., Mann J.F., McQueen M.J., Hess S., Paré G. (2019). A Mendelian randomization-based approach to identify early and sensitive diagnostic biomarkers of disease. Clin. Chem..

[9] Ritchie S.C., Liu Y., Lambert S.A., Teo S.M., Scepanovic P., Marten J., Zahid S., Chaffin M., Abraham G., Soranzo N. (2021). Integrative analysis of the plasma proteome and polygenic risk of cardiometabolic diseases. Nat. Metab..

[10] Ghodsian N., Abner E., Emdin C.A., Gobeil É., Taba N., Haas M.E., Perrot N., Manikpurage H.D., Gagnon É., Bourgault J. (2021). Electronic health record-based genome-wide meta-analysis provides insights on the genetic architecture of non-alcoholic fatty liver disease. Cell Rep. Med..

[11] Haycock P.C., Burgess S., Wade K.H., Bowden J., Relton C., Davey Smith G. (2016). Best (but oft-forgotten) practices: The design, analysis, and interpretation of Mendelian randomization studies. Am. J. Clin. Nutr..

[12] Grzych G., Vonghia L., Bout M.-A., Weyler J., Verrijken A., Dirinck E., Chevalier Curt M.J., Van Gaal L., Paumelle R., Francque S. (2020). Plasma BCAA changes in Patients with NAFLD are Sex Dependent. J. Clin. Endocrinol. Metab..

[13] Lovric A., Granér M., Bjornson E., Arif M., Benfeitas R., Nyman K., Ståhlman M., Pentikäinen M.O., Lundbom J., Hakkarainen A. (2018). Characterization of different fat depots in NAFLD using inflammation-associated proteome, lipidome and metabolome. Sci. Rep..

[14] Lim S., Taskinen M.R., Borén J. (2019). Crosstalk between nonalcoholic fatty liver disease and cardiometabolic syndrome. Obes. Rev..

[15] Jin R., Banton S., Tran V.T., Konomi J.V., Li S., Jones D.P., Vos M.B. (2016). Amino acid metabolism is altered in adolescents with nonalcoholic fatty liver disease—An untargeted, high resolution metabolomics study. J. Pediatrics.

[16] Lake A.D., Novak P., Shipkova P., Aranibar N., Robertson D.G., Reily M.D., Lehman-McKeeman L.D., Vaillancourt R.R., Cherrington N.J. (2015). Branched chain amino acid metabolism profiles in progressive human nonalcoholic fatty liver disease. Amino Acids.

[17] Andersson S.M., Salaspuro M., Ohisalo J.J. (1982). Metabolic basis of hypertyrosinemia in liver disease. Gastroenterology.

[18] Kimberly W.T., O’Sullivan J.F., Nath A.K., Keyes M., Shi X., Larson M.G., Yang Q., Long M.T., Vasan R., Peterson R.T. (2017). Metabolite profiling identifies anandamide as a biomarker of nonalcoholic steatohepatitis. JCI Insight.

[19] Wewer Albrechtsen N.J., Junker A.E., Christensen M., Hædersdal S., Wibrand F., Lund A.M., Galsgaard K.D., Holst J.J., Knop F.K., Vilsbøll T. (2018). Hyperglucagonemia correlates with plasma levels of non-branched-chain amino acids in patients with liver disease independent of type 2 diabetes. Am. J. Physiol. Gastrointest. Liver Physiol..

[20] Boulet M.M., Chevrier G., Grenier-Larouche T., Pelletier M., Nadeau M., Scarpa J., Prehn C., Marette A., Adamski J., Tchernof A. (2015). Alterations of plasma metabolite profiles related to adipose tissue distribution and cardiometabolic risk. Am. J. Physiol.-Endocrinol. Metab..

[21] Brennan A.M., Tchernof A., Gerszten R.E., Cowan T.E., Ross R. (2018). Depot-Specific Adipose Tissue Metabolite Profiles and Corresponding Changes Following Aerobic Exercise. Front. Endocrinol..

[22] Winther-Sørensen M., Galsgaard K.D., Santos A., Trammell S.A.J., Sulek K., Kuhre R.E., Pedersen J., Andersen D.B., Hassing A.S., Dall M. (2020). Glucagon acutely regulates hepatic amino acid catabolism and the effect may be disturbed by steatosis. Mol. Metab..

[23] Sookoian S., Castaño G.O., Scian R., Fernández Gianotti T., Dopazo H., Rohr C., Gaj G., San Martino J., Sevic I., Flichman D. (2016). Serum aminotransferases in nonalcoholic fatty liver disease are a signature of liver metabolic perturbations at the amino acid and Krebs cycle level1,2. Am. J. Clin. Nutr..

[24] Namjou B., Lingren T., Huang Y., Parameswaran S., Cobb B.L., Stanaway I.B., Connolly J.J., Mentch F.D., Benoit B., Niu X. (2019). GWAS and enrichment analyses of non-alcoholic fatty liver disease identify new trait-associated genes and pathways across eMERGE Network. BMC Med..

[25] Willer C.J., Li Y., Abecasis G.R. (2010). METAL: Fast and efficient meta-analysis of genomewide association scans. Bioinformatics.

[26] Burgess S., Thompson S.G., Collaboration C.C.G. (2011). Avoiding bias from weak instruments in Mendelian randomization studies. Int. J. Epidemiol..

[27] Winkler T.W., Day F.R., Croteau-Chonka D.C., Wood A.R., Locke A.E., Mägi R., Ferreira T., Fall T., Graff M., Justice A.E. (2014). Quality control and conduct of genome-wide association meta-analyses. Nat. Protoc..

[28] Kettunen J., Demirkan A., Würtz P., Draisma H.H., Haller T., Rawal R., Vaarhorst A., Kangas A.J., Lyytikäinen L.-P., Pirinen M. (2016). Genome-wide study for circulating metabolites identifies 62 loci and reveals novel systemic effects of LPA. Nat. Commun..

[29] Bowden J., Del Greco M.F., Minelli C., Davey Smith G., Sheehan N., Thompson J. (2017). A framework for the investigation of pleiotropy in two-sample summary data Mendelian randomization. Stat. Med..

[30] Verbanck M., Chen C.-Y., Neale B., Do R. (2018). Detection of widespread horizontal pleiotropy in causal relationships inferred from Mendelian randomization between complex traits and diseases. Nat. Genet..

[31] R Core Team (2010). R: A Language and Environment for Statistical Computing.

[32] Brunt E.M., Janney C.G., Di Bisceglie A.M., Neuschwander-Tetri B.A., Bacon B.R. (1999). Nonalcoholic steatohepatitis: A proposal for grading and staging the histological lesions. Am. J. Gastroenterol..

